# Si-rich Al_2_O_3_ films grown by RF magnetron sputtering: structural and photoluminescence properties versus annealing treatment

**DOI:** 10.1186/1556-276X-8-273

**Published:** 2013-06-07

**Authors:** Nadiia Korsunska, Larysa Khomenkova, Oleksandr Kolomys, Viktor Strelchuk, Andrian Kuchuk, Vasyl Kladko, Tetyana Stara, Oleksandr Oberemok, Borys Romanyuk, Philippe Marie, Jedrzej Jedrzejewski, Isaac Balberg

**Affiliations:** 1 V. Lashkaryov Institute of Semiconductor Physics, 45 Pr. Nauky, 03028, Kyiv, Ukraine; 2 CIMAP (CEA/CNRS/Ensicaen/UCBN), 6 Boulevard Marechal Juin, 14050, Caen, France; 3 Racah Institute of Physics, Hebrew University, 91904, Jerusalem, Israel

**Keywords:** Si-rich-Al_2_O_3_, Si nanocrystallites, Photoluminescence, XRD, Raman scattering

## Abstract

Silicon-rich Al_2_O_3_ films (Si_*x*_(Al_2_O_3_)_1−*x*_) were co-sputtered from two separate silicon and alumina targets onto a long silicon oxide substrate. The effects of different annealing treatments on the structure and light emission of the films versus *x* were investigated by means of spectroscopic ellipsometry, X-ray diffraction, micro-Raman scattering, and micro-photoluminescence (PL) methods. The formation of amorphous Si clusters upon the deposition process was found for the films with *x* ≥ 0.38. The annealing treatment of the films at 1,050°C to 1,150°C results in formation of Si nanocrystallites (Si-ncs). It was observed that their size depends on the type of this treatment. The conventional annealing at 1,150°C for 30 min of the samples with *x* = 0.5 to 0.68 leads to the formation of Si-ncs with the mean size of about 14 nm, whereas rapid thermal annealing of similar samples at 1,050°C for 1 min showed the presence of Si-ncs with sizes of about 5 nm. Two main broad PL bands were observed in the 500- to 900-nm spectral range with peak positions at 575 to 600 nm and 700 to 750 nm accompanied by near-infrared tail. The low-temperature measurement revealed that the intensity of the main PL band did not change with cooling contrary to the behavior expected for quantum confined Si-ncs. Based on the analysis of PL spectrum, it is supposed that the near-infrared PL component originates from the exciton recombination in the Si-ncs. However, the most intense emission in the visible spectral range is due to either defects in matrix or electron states at the Si-nc/matrix interface.

## Background

Silicon nanocrystallites (Si-ncs) attract considerable interest due to a significant transformation of optical and electrical properties in materials that contain them. These changes are caused by the quantum confinement effect [1-3]. Light-emitting Si-ncs embedded in dielectric hosts have potential applications in optoelectronic devices because of their compatibility with the existing manufacturing infrastructure for silicon integrated circuits.

Among different dielectric materials, silicon oxide is the most addressed as a host for Si-ncs [4,5]. During the last decades, the properties of Si-nc-SiO_2_ systems have been widely investigated. Bright luminescence in a wide spectral range at room temperature originates from recombination of excitons in Si-ncs; the variation of their sizes allows tuning of the emission wavelength from the blue to the near infrared [3-6]. In addition to the attractive photoluminescence property, these materials can be used for a new generation of solar cells [7]. Furthermore, Si-ncs embedded in dielectric matrices have regained interest as candidates for non-volatile memory applications [8]. However, because of the downscaling of microelectronic devices, silicon oxide met its limit as a gate material due to high leakage current. In this regard, high-k dielectrics such as ZrO_2_, HfO_2_, and Al_2_O_3_ are considered as promising gate dielectrics due to the lower equivalent oxide thickness. Also, Si-ncs embedded in such high-k host offer a wider application for non-volatile memories due to the higher performance of the corresponding devices [9,10].

From the photonic application viewpoint, Al_2_O_3_ is an interesting host material for optical communication. The relatively higher refractive index of Al_2_O_3_ (1.73 at 1.95 eV) in comparison with that of SiO_2_ (1.46 at 1.95 eV) at similar bandgap energies allows better light confinement, making compact device structures possible. Indeed, alumina-based waveguides that are very important for optical communications have been developed [11,12]. Alumina co-doped with Si-ncs and Er^3+^ ions is more promising than similarly co-doped silica due to higher solubility of Er^3+^ ions in alumina host. However, in spite of promising properties, Si-nc-Al_2_O_3_ materials were not well addressed.

Several approaches have been used to form Si-ncs in amorphous and/or crystalline Al_2_O_3_. Most known methods are Si ion implantation [13,14] and electron beam evaporation followed by subsequent high-temperature annealing as well as laser ablation [15]. For these systems, the successful Si-nc formation was already demonstrated. However, in spite of the relative simplicity of magnetron sputtering technique and its wide application for the fabrication of Si-rich SiO_2_ materials [5,8], only few groups applied this method for deposition of Si-rich alumina [16].

The present paper reports the fabrication of Si-rich Al_2_O_3_ films with different Si content by magnetron co-sputtering and the effect of post-deposition processing on the structural and luminescent properties of these materials.

## Methods

The Si-rich Al_2_O_3_ films were deposited by radio frequency (RF) magnetron co-sputtering of two separate 2-in. targets (pure Si and Al_2_O_3_) on a long quartz substrate at room temperature. The use of long substrate allowed the variation of the composition along film length in a single deposition run. The length and the width of deposited film were 140 and 4 mm, respectively. The distance between the targets and the substrate was fixed at 64 mm. The background vacuum in the chamber was about 1 × 10^−5^ Pa prior to the deposition with the pure argon plasma. The RF power applied on Si and Al_2_O_3_ targets were 40 and 80 W, respectively. Apart from Si-rich Al_2_O_3_ films, pure Si and pure Al_2_O_3_ were deposited at the same conditions from one target only. The deposition time was 250 min for each deposition run. The as-deposited original films were cut then to smaller (1 cm in length) segments (called hereafter as samples) to simplify the investigation of their properties.

To study the chemical composition of the films, their refractive index and thickness, the spectroscopic ellipsometry measurement was performed by means of a Jobin-Yvon ellipsometer (UVISEL, HORIBA Ltd., Kyoto, Japan), where the incident light was scanned in the range of 1.5 to 4.5 eV under an incident angle of 66.3°. The fitting of the experimental data was performed using DeltaPsi2 software (HORIBA Ltd., Kyoto, Japan) [17] and allowed to get information about variation of refractive index and thickness along the film length. Additionally, the film thickness was controlled by means of a Dektak 3030 Profilometer (Veeco, Plainview, NY, USA). The thickness obtained by both methods was found to increase gradually from about 660 nm (Al_2_O_3_ side) up to about 1,280 nm (Si side).

To form Si-ncs in the alumina host, two post-fabrication treatments were applied. The former was a conventional annealing (CA) in a horizontal furnace at 1,150°C for 30 min in a nitrogen flow. Another one was a rapid thermal annealing (RTA) at 1,050°C for 1 min either in air or nitrogen atmosphere.

To investigate the evolution of the microstructure and the luminescent properties of the films, we applied a Horiba Jobin-Yvon T-64000 Raman spectrometer (HORIBA Ltd., Kyoto, Japan) equipped with confocal microscope and automated piezo-driven XYZ stage. The measurements were performed at the center of each segment. The micro-Raman scattering (μ-RS) and micro-photoluminescence (μ-PL) spectra were detected in 100- to 900-cm^−1^ and in 500- to 900-nm spectral ranges, respectively. A 488.0-nm line of Ar-Kr ion laser was used as the excitation source. The laser power on the sample surface was always kept below 5 mW to obtain the best signal-to-noise ratio, preventing a laser heating of the investigated sample. The spectral resolution of the spectrometer was less than 0.15 cm^−1^. X-ray diffraction (XRD) in our study was carried out using Philips X’Pert-MRD diffractometer (PANalytical B.V, Almelo, The Netherlands) with Cu Kα radiation (*λ* = 0.15418 nm) in a grazing geometry. The structural investigations were performed at 300 K, whereas the PL was measured at 300 and 80 K.

## Results and discussion

### Spectroscopic ellipsometry analysis

It is known that spectroscopic ellipsometry is a fast, sensitive, and non-destructive method for thin-film characterization [18-20]. It requires no special environments and can be easily integrated into semiconductor processing. The spectral dependencies of ellipsometric angles (Ψ and Δ) are defined from the fundamental equation of ellipsometry $\bar{r_{p}}/\bar{r_{s}}=\tan\Psi \exp\mathrm{i\Delta}$, where $\bar{r_{p}}$ and $\bar{r_{s}}$ are the complex reflection coefficients for parallel and perpendicular polarizations of light, respectively. These dependencies of Ψ and Δ can be fitted with appropriate modeling approaches to extract the film thickness and optical constants (refractive index, *n*, and extinction coefficient, *k*) based on the best fit between experimental and simulated spectra [18,20].

To fit of ellipsometry data, the dispersion law was chosen based on the Forouhi-Bloomer model elaborated for amorphous semiconductor and insulating materials [21] using an improved parameterization [22]. The dispersion formulae for *n* and *k* were given as follows:

(1) $$\begin{array}{l} n\left(\omega \right)=n_{\infty }+\frac{B_{i}\cdot \left(\omega -\omega_{i}\right)+C}{{\left(\omega -\omega_{i}\right)}^{2}+\Gamma_{i}^{2}},\\ k\left(\omega \right)=\left\{\begin{array}{c} \frac{f_{i}\cdot {\left(\omega -\omega_{g}\right)}^{2}}{{\left(\omega -\omega_{i}\right)}^{2}+\Gamma_{i}^{2}},\;\omega >\omega_{g}\;\\ 0,\omega \leq \omega_{g} \end{array}\right), \end{array}$$

where $B_{i}=f_{i}\cdot \left(\Gamma_{i}^{2}-{\left(\omega_{i}-\omega_{g}\right)}^{2}\right)\Gamma_{i}\;,C=2\cdot f_{i}\cdot \Gamma_{i}\cdot \left(\omega_{i}-\omega_{g}\right),$*n*_∞_ is a refractive index at high frequency, *f*_*i*_ is an oscillator strength, *Γ*_*j*_ is an amortization factor, *ω*_*i*_ and *ω*_*g*_ are frequencies of free oscillator. Two dependences, *I*_s_ = *I*⋅sin2Ψ⋅sinΔ and *I*_c_ = *I*⋅sin2Ψ⋅cosΔ, where $I=\frac{E_{0}}{4}\left({\left|r_{p}\right|}^{2}+{\left|r_{s}\right|}^{2}\right)$ and *E*_0_ is the amplitude of electric field of incident light, were fitted.

**Figure 1:**
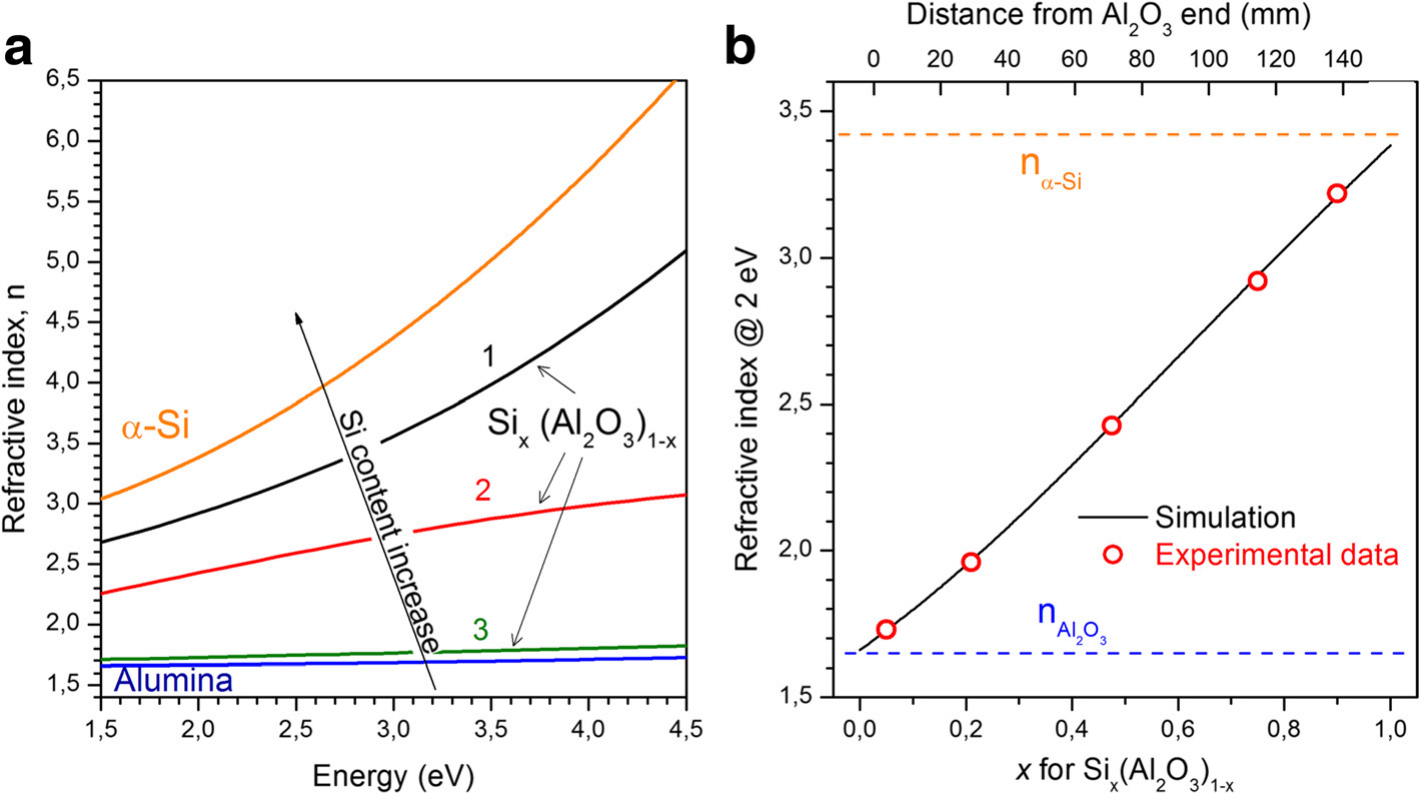
**Refractive index variation for Si-rich Al**_**2**_**O**_**3**_**, pure amorphous Si, and Al**_**2**_**O**_**3 **_**films.** (**a**) Refractive index variation for pure amorphous Si and Al_2_O_3_ films as well as Si-rich-Al_2_O_3_ samples with different Si content, *x* = 0.50 (1), 0.22 (2), and 0.05 (3). (**b**) Simulated variation of the refractive index, *n*, taken at 2 eV, versus Si content (*x*) in Si-rich Al_2_O_3_ (solid line). The circle symbols of this curve represent experimental *n* values, used for estimation of the *x* values.

The spectral dependencies of refractive indexes for as-deposited films grown from one target only (either pure Si or Al_2_O_3_ films) and from both targets (Si-rich Al_2_O_3_ one) are shown in Figure 1a. It is seen that the *n* values obtained for pure Si and Al_2_O_3_ films are in the agreement with the data of Ref. [23,24]. This means that magnetron sputtering approach allows deposition of the materials with the same stoichiometry as initial target.

As for Si-rich Al_2_O_3_ films grown from both targets, their dispersion curves are found to be between the curves corresponded to pure Al_2_O_3_ and amorphous silicon. They demonstrate gradual shift toward the dependence for amorphous Si with Si content increase (Figure 1a). This means that the film can be considered rather as a mixture of Al_2_O_3_ and Si (or SiO_*x*_ with *x* < 1), then a mixture of Al_2_O_3_ with SiO_2_ similar to the case described for Si-rich HfO_2_ films [20]. All the films were found to be amorphous as confirmed by Raman scattering and XRD data (see below). Thus, hereafter, we consider our Si-rich Al_2_O_3_ film as an effective medium, which macroscopic properties are determined by the relative fractions of Si and Al_2_O_3_, i.e., Si_*x*_(Al_2_O_3_)_1−*x*_.

To predict the variation of refractive index *n* versus *x*, the Bruggeman effective medium approximation was used based on the approach described in [25]. In this case, the variation of dielectric function (i.e., refractive index) is defined by the following two equations:

(2) $$\sum_{i}v_{i}\frac{\varepsilon_{i}-\varepsilon }{\varepsilon_{i}+2\varepsilon }=0,$$

(3) $$\sum_{i}v_{i}=1,$$

where *ε*_*i*_ and *ν*_*i*_ are the complex optical dielectric function and volume fraction for the *i*th component, respectively; *ν* is the effective dielectric function corresponding to the measured value for the film. The results of this simulation are presented for the *n* taken at 2.0 eV (Figure 1b). The dots on this curve correspond to the experimental *n* values obtained by fitting of ellipsometry data (taken also at 2.0 eV). This approach allows rough estimation of the *x* variation along the film length (Figure 1b). Taking into account Eqs. (2) and (3) and the values of corresponding refractive indexes (Figure 1a), the relative fraction of Si phase was found to vary from *x* ≈ 0.92 (*n* = 3.22 ± 0.01; Si-rich side) to *x* ≈ 0.05 (*n* = 1.73 ± 0.01; Si-poor side) (Figure 1b). It should be noted that for *x* > 0.7, our films grown from Si and Al_2_O_3_ targets can be considered rather as Al_2_O_3_-rich Si films than Si-rich alumina. In this regard, hereafter, the samples with *x* < 0.7 will be only analyzed.

### Raman scattering spectra

#### As-deposited films

Since important information on the structure of amorphous/nanocrystalline silicon can be obtained from its Raman scattering spectra [26,27], we investigated these spectra for as-deposited and annealed films versus *x*.

It is known that for amorphous Si (a-Si), all phonon modes of the transverse acoustic (TA), longitudinal acoustic (LA), longitudinal optical (LO), and transverse optical (TO) modes are active due to the lack of translational invariance. The Raman spectrum from a-Si is, then, a measure of the density of vibration states that are modified substantially by small changes in the short-range order [26]. It has been shown that the full width at half maximum (*Γ*_TO_), the peak position of the TO phonon mode (*ω*_TO_), and the ratio of the intensities of TO (*I*_TO_) and TA (*I*_TA_) modes, (I_TA_/I_TO_), depend almost linearly on the average bond-angle variation (ΔΘ) in an a-Si network [27]:

(4) $${Г}_{\mathrm{ТО}/}{}_{2}=3\Delta \Theta +7.5$$

(5) $$\omega_{\mathrm{TO}}=-2.5\Delta \Theta +505.5$$

(6) $$I_{\mathrm{TA}/\mathrm{TO}}=0.0078\Delta \Theta +0.0606.$$

**Figure 2:**
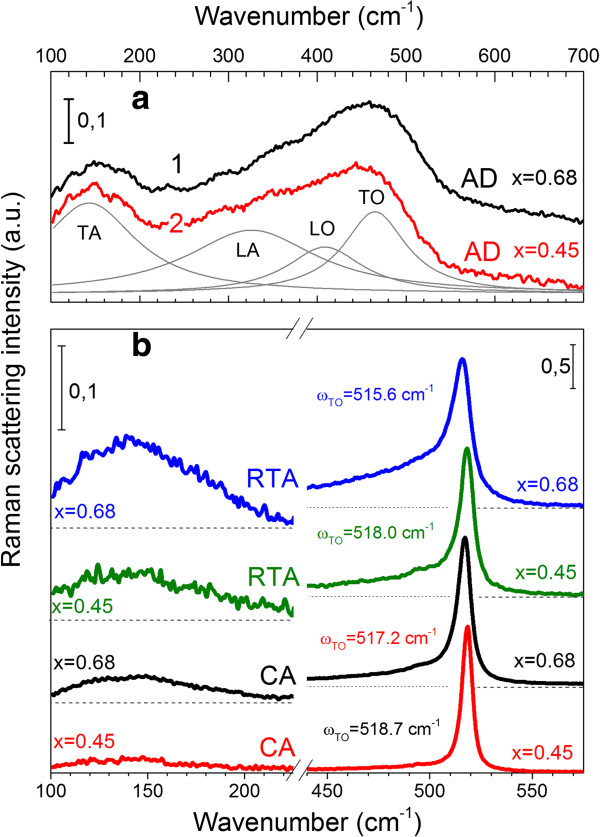
**Micro-Raman spectra of as-deposited, RTA-, and CA-treated Si-rich Al**_**2**_**O**_**3 **_**films.** (**a**) Micro-Raman spectra of as-deposited Si-rich Al_2_O_3_ films with *x* = 0.68 (1) and *x* = 0.45 (2). The deconvolution of curve 2 to four Si-phonon bands is also present. The spectra are offset for clarity. (**b**) Variation of micro-Raman spectra after RTA and CA treatments on the same samples.

Raman scattering spectra were obtained for the films with *x* ≥ 0.38, whereas for lower *x* values the signal was not detected. As Figure 2a shows, the first-order μ-RS spectra consist of two distinct broad bands peaked at 140 to 160 cm^−1^ and 460 to 470 cm^−1^ (curves 1, 2). These spectra are typical for amorphous silicon and can be described as overlapping of four bands related to acoustic and optical Si phonon modes: transverse and longitudinal acoustic (TA and LA) phonons as well as longitudinal and transverse optical (LO and TO) modes. The deconvolution of the spectrum for sample with *x* = 0.45 is shown in Figure 2a. It is worth to note that the peak position of TO phonon mode is shifted toward the lower wave numbers (*ω*_ТО_ ≈ 460 cm^−1^) with the respect to the peak position of TO phonon observed usually in the spectra of ‘relaxed’ a-Si (*ω*_ТО_ ≈ 480 cm^−1^) (Figure 2, curve 2).

This *ω*_ТО_ shift indicates ‘unrelaxed’ microstructure of a-Si in our samples due to either point defects (caused a ΔΘ distortion) or tensile strain field [26,27]. Based on Eqs. (4) and (5), the ΔΘ value was found to be ΔΘ ≈ 20° (*x* = 0.45) and ΔΘ ≈ 18° (*x* = 0.68) that exceeds significantly the ΔΘ values obtained for ‘relaxed’ a-Si (about ΔΘ = 7° to 11° [26,27]). This is an evidence of the significant short-range disorder in a-Si phase in our samples, which can result from numerous point defects or small size of a-Si clusters. At the same time, the ΔΘ values obtained from Eq. (6) are much higher: ΔΘ ≈ 70° (*x* = 0.45) and ΔΘ ≈ 63° (*x* = 0.68). This can be explained by significant middle-range disorder that can be caused by the contribution of elastic strains [26,27]. In our case, they are tensile since the *ω*_ТО_ shifts to the lower wavenumbers.

The observation of Raman spectrum of a-Si in the as-deposited films with *x* ≥ 0.38 is the evidence of a-Si clusters' formation during film deposition. Meanwhile, when *x* < 0.42, the intensity of the Raman spectra decreases significantly; the *Г*_TO_ and the *I*_TA/TO_ values increase, and the *ω*_ТО_ shifts to the lower frequencies. This testifies to disorder enhancement and can be caused by the decrease the sizes and number of a-Si clusters.

#### Annealed films

After either CA or RTA treatment, a narrow and high-energy peak is observed, indicating the formation of Si nanocrystallites. For both treatments, with the *x* decrease the peak position (*ω*_ТО-Si-nc_) slightly shifts toward the higher wavenumbers accompanied by the decrease of its full width at half maximum (*Γ*_TO-Si-nc_) (Figure 2b). It is observed in the range of *ω*_ТО-Si-nc_ = 517.3 to 518.6 cm^−1^ for CA samples and *ω*_ТО-Si-nc_ = 513.6 to 516.0 cm^−1^ for RTA samples. At the same time, for the samples with the same *x* values, Raman peak position is essentially controlled by annealing conditions: the increase of temperature and duration results in its high-wavenumber shift (about 5 cm^−1^) (Figure 2b). Observed variation of the *ω*_ТО-Si-nc_ and *Γ*_TO-Si-nc_ versus the *x* (Figure 2b) contradicts to that expected for quantum confinement effect, because with the *x* decrease, the Si-nc sizes have to reduce, demonstrating the shift of *ω*_ТО-Si-nc_ toward the lower wavenumbers and the increase of the *Γ*_TO-Si-nc_[28].

As one can see from Figure 2b, besides Si-nc-related peak, the features in the ranges from 100 to 180 cm^−1^ and 420 to 480 cm^−1^ are present. This means that all annealed samples contain the amorphous silicon phase, which amount increases with the *x* rise. This can explain the shift of Raman peak position toward lower wavenumbers for higher *x* values.

It is worth to note that the *ω*_ТО-Si-nc_ for the Si-nc formed in sapphire at 700°C to 1,050°C is observed in the range from 520 to 525 cm^−1^[13] and is shifted to the higher-energy side with respect to peak position of intrinsic c-Si. This indicates the Si-nc in sapphire are under the compressive stress [13]. In contrast in our samples, the *ω*_ТО-Si-nc_ is shifted to the lower wavenumbers (below 519 cm^−1^). This ‘red’ shift can be caused either by the quantum confinement effect or by the tensile strain between the Si-rich Al_2_O_3_ film and the quartz substrate. Going further, based on the XRD data obtained for these samples (see below), we can explain this *ω*_ТО_ shift by the strain between the film and the substrate that is in agreement with the μ-RS data obtained for as-deposited samples. It should be noted that most probable explanation of the smaller shift of the *ω*_ТО-Si-nc_ value after CA treatment in comparison with that after RTA one is the relaxation of tensile stress due to longer time and higher temperature of CA treatment.

The presented results show that the *ω*_ТО_ peak position for annealed samples does not allow correct estimation of the variation of Si-nc sizes because of mechanical stress and presence of amorphous Si phase. Thus, an additional study of structural properties of the samples was performed by means of X-ray diffraction method.

### XRD diffraction study

**Figure 3:**
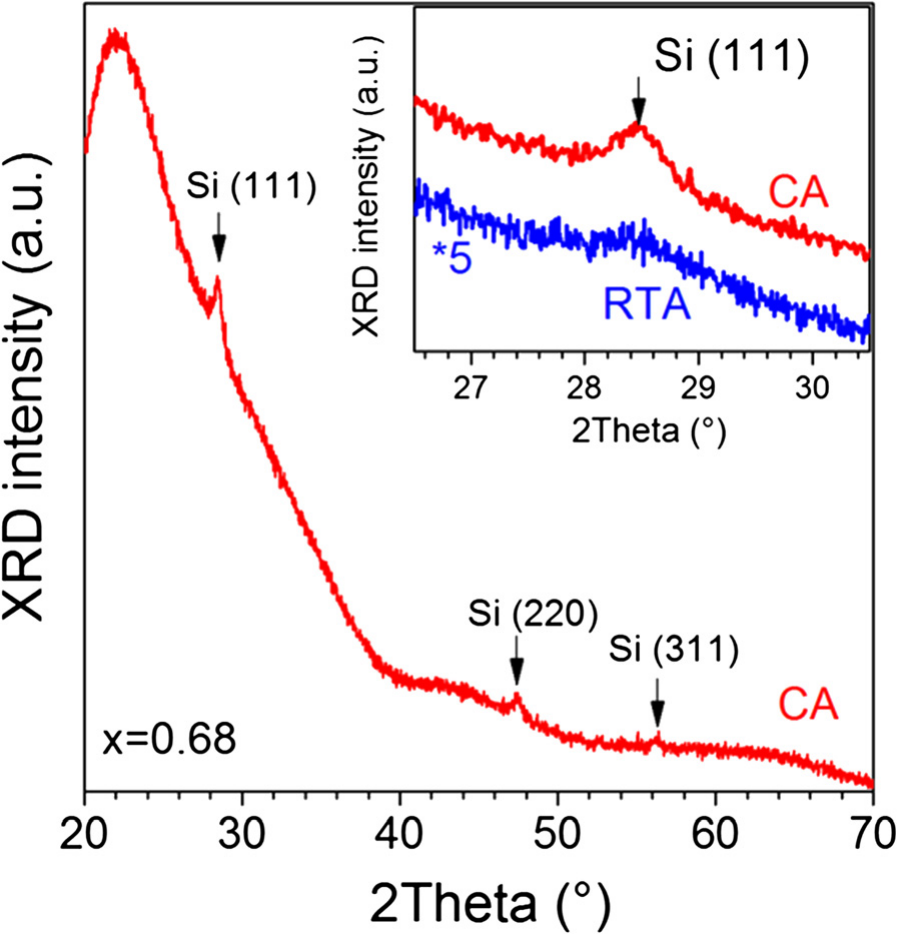
**The XRD patterns of the samples submitted to CA and RTA treatments.** XRD pattern for a sample with *x* = 0.68 after CA treatment at 1,150°C for 30 min in nitrogen flow. The inset shows the expanded presentation of the (111) Si peak for CA and RTA samples with *x* = 0.68. RTA treatment was performed at 1,050°C for 1 min in nitrogen flow.

The information on the Si-nc sizes was obtained from an XRD study of the samples with *x* ≥ 0.5. Figure 3 shows the XRD pattern of CA sample with *x* = 0.68, where several peaks correspond to beam diffraction from the Si crystallographic planes at 2*Ө* = 28.4° (111), 2*Ө* = 47.3° (220), and 2*Ө* = 56.2° (311). The intensity of XRD peaks decreases with the *x* decrease, and for the *x* < 0.5, they are not detectable.

The RTA samples showed the same Si-related XRD peaks, but they are broader (Figure 3, inset). There was not significant effect of the atmosphere of the RTA treatment (either air or nitrogen) on XRD patterns. No diffraction peak from crystalline Al_2_O_3_ was detected which indicates that the Si-ncs are embedded in an amorphous matrix.

The mean size of the Si-ncs (<*d*_Si_>) was calculated using the Scherer formula. It was found that for *x* = 0.5 to 0.68, they did not depend practically on the *x* values but were affected by the treatment conditions. The estimation showed that <*d*_Si_> ≈ 14 nm for CA samples and <*d*_Si_> ≈ 5 nm for RTA samples. However, it does not exclude the existence of the smaller crystallites in the samples.

The comparison of the XRD data (Figure 3) and the μ-RS spectra (Figure 2) obtained for the same annealed samples showed that the ‘red’ shift of the Si-related TO phonon in the μ-RS spectra (to about 517.3 cm^−1^) is observed for the Si-nc with <*d*_Si_> ≈ 14 nm when a quantum confinement effect is negligible. This allows concluding that the tensile stress between the film and the substrate affects significantly the peak position of the TO phonon in Raman scattering spectra (Figure 2).

### Light-emitting properties of the samples

#### As-deposited films

**Figure 4:**
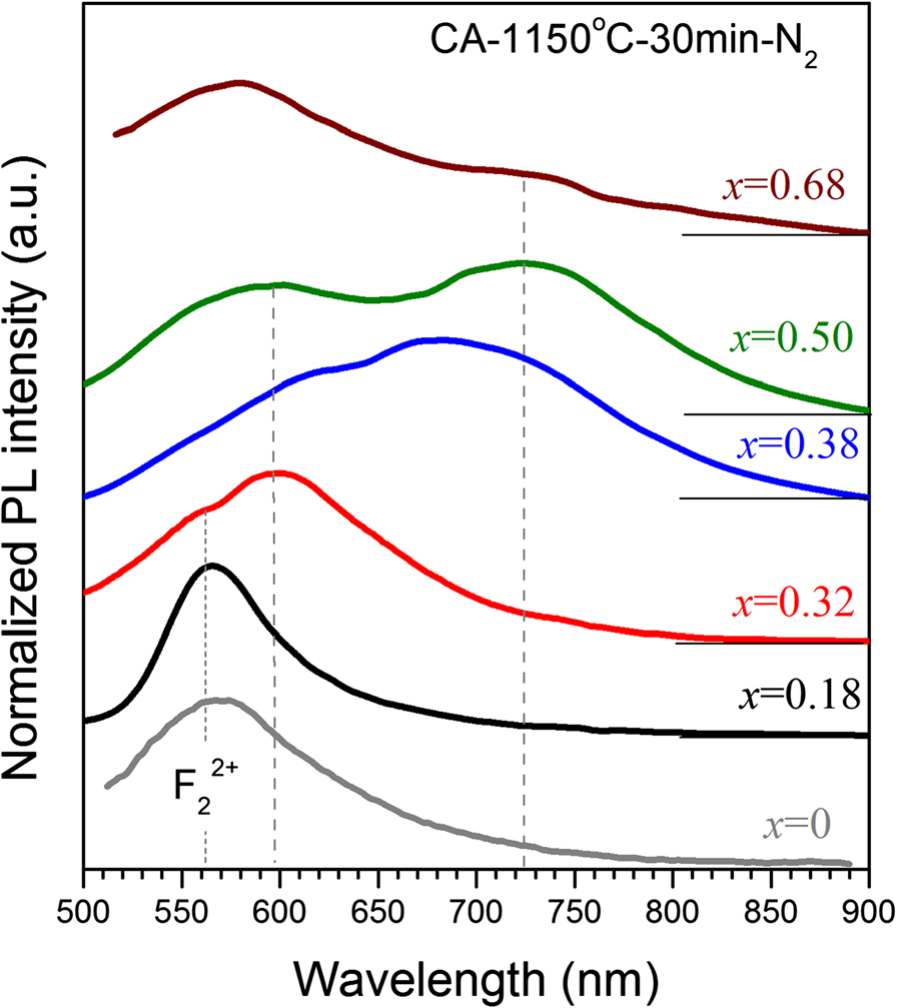
**PL spectra of the samples with different *x *values submitted to conventional annealing.** This treatment was performed at 1,150°C for 30 min in N_2_ flow. The *x* values are mentioned in the figure. The spectrum for *x* = 0 corresponds to the emission of Al_2_O_3_ film.

PL emission from as-deposited samples with x = 0.5 to 0.18 shows only the peak at about 560 nm (Figure 4) which is also observed in pure Al_2_O_3_ film (Figure 4, curve at *x* = 0) and can be assigned to F_2_^2+^ centers in Al_2_O_3_[29]. At the same time, either CA or RTA treatment yields visible PL emission in wider spectral range.

#### PL after conventional annealing treatment

Figure 4 represents the PL spectra of CA samples measured at 300 K. These spectra contain two broad PL bands, whose maxima are observed at 575 to 600 nm and 700 to 750 nm. In the samples with *x* = 0.5 to 0.68, these PL bands are well separated, whereas for the films with *x* = 0.38, they are overlapped significantly (Figure 4). For *x* = 0.18 to 0.32, the PL intensity of the first band (peaked at 575 to 600 nm) appears to exceed essentially the magnitude of the second PL band (centered at 700 to 750 nm), while the contribution of the second band is more pronounced for the samples with *x* = 0.38 to 0.68. As Figure 4 shows the first band consists of two components with maxima positions at about 560 and about 600 nm. The former one (about 560 nm) is clearly seen in the sample with *x* = 0.18 and is similar to PL emission from F_2_^2+^ centers in Al_2_O_3_. Furthermore, it presents in other spectra also, testifying to the incorporation of Si inclusions into Al_2_O_3_ matrix. At the same time, both components are strongly overlapped in the samples with *x* = 0.32 to 0.68 (Figure 4).

#### PL after rapid thermal annealing

The RTA treatment of the samples in nitrogen atmosphere results in the weak PL emission, whereas the RTA treatment in air causes a much brighter visible emission (Figure 4) that is in agreement with the data of Ref. [16]. The broad PL spectrum can be considered as overlapping of several PL bands (similar to the case of CA treatment).

**Figure 5:**
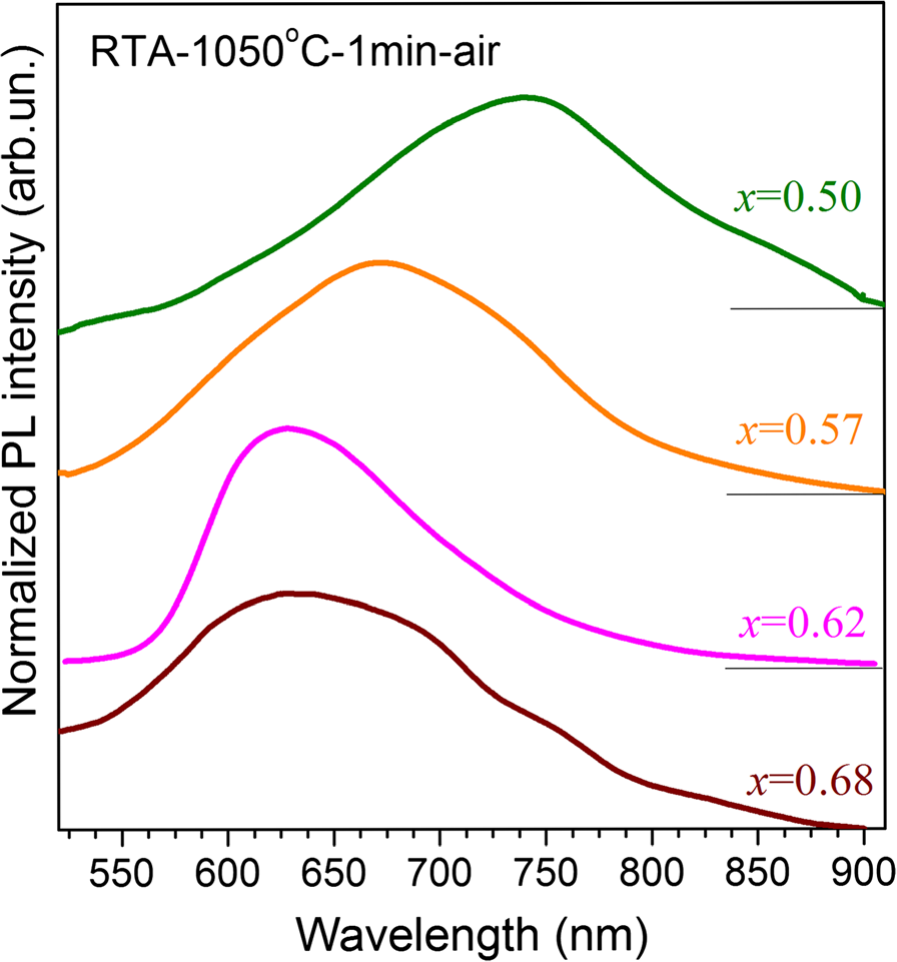
**PL spectra of the samples with different *x *values after RTA treatment.** This annealing was performed at 1,050°C for 1 min in air.

The samples with *x* = 0.5 to 0.68 showed only one broad PL which peak position shifts to long wavelength side with the *x* decrease (Figure 5). This can be a result of the overlapping of different PL components similar to that observed for CA-treated samples (Figure 4). Besides, the shoulder (or tail) can be also observed in the 825- to 900-nm range (Figure 5).

#### PL spectra of annealed samples versus temperature of measurement

To elucidate the origin of PL emission from the films investigated, the PL spectra were measured also at 80 K. It should be expected that peak position and intensity of PL bands related to defects in oxide matrixes will not change in the intensity and peak position under cooling down to 80 K because of deep-level-related intra-defect transition. In fact, the most oxide defects demonstrate such PL behavior in the 80 to 300 K range. In contrast, the PL band, related to exciton recombination in quantum confinement Si-ncs, has to demonstrate the shift of its peak position to higher-energy side (up to approximately 41 meV) due to Si bandgap increase [30,31] accompanied by the increase of PL intensity [32]. However, it is worth to note that the appearance of the strains as well as their sign (tensile or compressive) results either in the increase or in the decrease of this PL shift [33].

**Figure 6:**
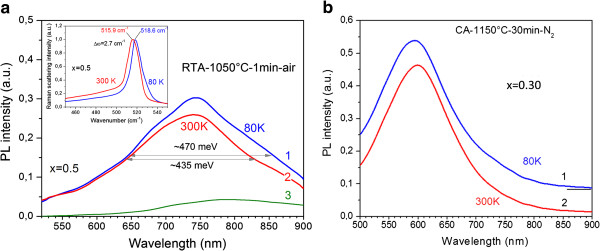
**PL spectra of RTA-treated (a) and CA-treated (b) samples versus temperature of measurement.** The spectra were detected at 80 K (curves 1) and 300 K (curves 2) with *x* = 0.50 (**a**) and 0.32 (**b**). The spectra in (**b**) are shifted vertically for clarity.

The investigation of Raman scattering spectra at low temperature shows that the peak position of Si-nc-related TO phonon shifts to higher energy side (about 2.7 cm^−1^) (Figure 6a, inset). At the same time, for the bulk Si, this shift is about 4.5 cm^−1^[34]. This means that the cooling of the samples investigated results in the increase of tensile stress in Si-ncs leading to the low-energy shift of corresponding TO phonon by 1.8 cm^−1^. This hides the expected high-energy shift of Si-nc TO phonon under cooling. Such stresses are due to the difference in thermal expansion coefficients of Al_2_O_3_ (5.4 × 10^−6^ K), Si (3 × 10^−6^ K), and SiO_2_ (0.77 to 1.4 × 10^−6^ K). In particular, with cooling, Al_2_O_3_ will compress much more than SiO_2_. Thus, SiO_2_ substrate will stretch Al_2_O_3_ film, and additional tensile stress in Al_2_O_3_ will appear under cooling. At the same time, Al_2_O_3_ host has to compress Si-ncs. Based on Raman scattering data, we estimated the relative deformation in Si-nc appeared under cooling. It was found biaxial tensile deformation which is about 0.15%. Taking into account the results of Ref. [35], one can see that such deformation causes the narrowing of Si bandgap by 22 meV. Thus, as consequence, the shift of the peak position of Si-nc-related PL band has to be about 19 meV only. Such a shift for the broad featureless PL bands, observed in our experiment, can be negligible. Therefore, hereafter, the variation of PL intensity only will be considered.

As one can see from Figure 6b, in CA samples with the x ≤ 0.32, where PL spectrum is dominated by one band with peak position at 575 to 600 nm, its peak position and intensity do not depend on temperature. Thus, one can conclude that this emission in our Si-rich Al_2_O_3_ films originates from the defects. Such a band was observed in Si-rich Al_2_O_3_ materials [36,37] as well as in Si-rich SiO_2_ samples [5]. In the former case, it was ascribed to F_2_^2+^ centers in Al_2_O_3_, whereas in the latter case to E′ and NBOHC defects in SiO_2_. Thus, this emission can be ascribed to the defects located near Si-nc/host interface (i.e., in the shell that covered these Si-ncs). This shell can consist of both alumina and silica [13,16].

The PL spectra of RTA samples are complex, and they have complicated temperature behavior. As one can see from Figure 6a, PL peak position, observed for sample with *x* = 0.5 at 700 to 750 nm, is independent on temperature, whereas the intensity of short-wavelength wing (500 to 650 nm) does not change with cooling (Figure 6a). At the same time, a broadening of PL band toward longer wavelengths and slight increase of its intensity in maximum are observed. The independence of the intensity of short-wavelength component (500 to 650 nm) is similar to the data obtained for CA samples that allows its ascribing to the radiative recombination of carriers via host defects.

Since PL spectrum of RTA samples contains several overlapped PL components with very weak features, its deconvolution can be hardly performed. Thus, we used the subtraction of the PL spectrum detected at 300 K from that measured at 80 K. It is seen that PL intensity in the 780- to 900-nm spectral range increases with cooling (Figure 6a, curve 3). The most probable reason for this increase is the rise of the contribution of carrier recombination in Si-ncs to the PL spectrum that is in agreement with the data of Ref. [5,32] (Figure 6a). At the same time, the PL component peaked at 700 to 750 nm can be attributed to the defects located at Si-nc/matrix interface because slight increase of its maximum magnitude is apparently due to overlapping with near-infrared component which intensity increases with cooling (Figure 6a, curve 3).

Based on the PL results, one can conclude that the main contribution to the PL spectra in our samples is given by the carrier recombination through different defects. The high concentration of interface and matrix defect (in particular, the high intensity of PL band at 700 to 750 nm) obviously hinders the observation of exciton recombination.

## Conclusions

The effect of annealing treatment on structural and light emission properties of Si phase-rich Al_2_O_3_ films with different Si contents was investigated. The formation of amorphous Si clusters upon deposition process was observed for the films with *x* ≥ 0.38. The annealing results in the formation of Si crystallites whose mean size depends on the type of post-deposition treatment. The conventional annealing of the samples with *x* = 0.5 to 0.68 causes the formation of Si-ncs with the mean size of about 14 nm, whereas similar samples submitted to rapid thermal annealing show the presence of Si-ncs with sizes of about 5 nm. Two main broad PL bands were observed in the 500- to 900-nm spectral range with peak positions at 575 to 600 nm and 700 to 750 nm as well as near-infrared tail. The low-temperature measurements revealed that the first PL band was unchanged with cooling, while the slight increase of maximum intensity of the second one was obviously due to overlapping with near-infrared band. Such behavior of visible PL bands differs from that expected for quantum confined Si-ncs that allowed ascribing them to interface and/or matrix defects. At the same time, the analysis of PL spectrum shape allows ascribing the near-infrared PL component (780 to 900 nm) to the exciton recombination inside Si-ncs.

## Competing interests

The authors declare that they have no competing interests.

## Authors’ contributions

NK designed and coordinated the study as well as, together with LK, prepared the draft of the manuscript. JJ fabricated the samples investigated. JJ and TS performed conventional annealing treatment. VS and OK carried out μ-Raman and μ-PL characterization. VK and AK performed XRD measurements. OO and BR performed RTA treatment and thickness measurement. PM and LK performed spectroscopic ellipsometry study and fit the data. NK, LK, IB and VS corrected the manuscript till its final version. All authors read and approved the final manuscript.
